# Supplementary material for: Dietary Supplementation of 25-Hydroxycholecalciferol Improves Livability in Broiler Breeder Hens-Amelioration of Cardiac Pathogenesis and Hepatopathology
Source: Animals (Basel). 2019 Oct 8;9(10):770. doi: 10.3390/ani9100770 (PMC6826734; doi:10.3390/ani9100770)
Supplement: Supplementary file 1 [file animals-09-00770-s001.pdf]

Supporting information for online publication

Table S1. Effect of dietary supplementation of 25-OH-D3 on the incidence of cardiac pathological morphology of broiler breeder hens provided with restricted or ad libitum feed intake

Hens died by sudden death[1,3] (hearts of dead hens)	Restriction	Restriction +25-OH-D3	Ad libitum	Ad libitum +25-OH-D3
**Type 1; concentric hypertrophy (total)**	11/19	7/12	47/78	34/57
**26-29 wks**	1/1	0/1	3/4	1/2
**30-35 wks**	1/2	1/1	15/21	10/14
**36-47 wks**	4/6	3/6	16/30	15/24
**after 48 wks**	5/10	3/4	13/23	8/17
**Type 2; dilation (eccentric)(total)**	2/19	1/12	25/78	17/57
**26-29 wks**	0/1	0/1	0/4	0/2
**30-35 wks**	0/2	0/1	5/21	3/14
**36-47 wks**	0/6	0/6	11/30	7/24
**after 48 wks**	2/10	1/4	9/23	7/17
**Type 3; pericardial effusion (total)**	4/19	2/12	25/78	16/57
**26-29 wks**	1/1	0/1	2/4	1/2
**30-35 wks**	0/12	0/1	7/21	5/14
**36-47 wks**	2/6	1/6	8/30	5/24
**after 48 wks**	1/10	1/4	7/23	5/17
**Type 4; ascites (total)**	1/19	1/12	8/78	5/57
**26-29 wks**	0/1	0/1	0/4	0/2
**30-35 wks**	0/2	0/1	2/21	1/14
**36-47 wks**	1/6	1/6	4/30	4/24
**after 48 wks**	0/10	0/4	2/23	0/17
**Type 5; infarction damage (total)**	4/19	4/12	17/78	13/57
**26-29 wks**	1/1	1/1	2/4	0/2
**30-35 wks**	2/2	1/1	5/21	5/14
**36-47 wks**	1/6	1/6	4/30	6/24
**after 48 wks**	0/10	0/4	6/23	2/17
**Type 6; atrium rupture trauma (total)**	1/19	0/12	4/78	2/57
**26-29 wks**	0/1	0/1	0/4	0/2
**30-35 wks**	0/2	0/1	1/21	0/14
**36-47 wks**	0/6	0/6	3/30	1/24
**after 48 wks**	1/10	0/4	1/23	1/17

Hens sampled for tissue collection[2,3] (hearts of sampling hens)	Restriction	Restriction +25-OH-D3	Ad libitum	Ad libitum +25-OH-D3
**Type 1; concentric hypertrophy (total)**	2/18	2/18	7/18	5/18
**at age of 29 wks**	0/4	0/4	1/4	0/4
**at age of 35 wks**	1/7	1/7	3/7	2/7
**at age of 47 wks**	1/7	1/7	3/7	3/7
**Type 2; dilation (eccentric)(total)**	2/18	2/18	6/18	4/18
**at age of 29 wks**	0/4	0/4	0/4	0/4
**at age of 35 wks**	1/7	1/7	3/7	2/7
**at age of 47 wks**	1/7	1/7	3/7	2/7
**Type 3; pericardial effusion (total)**	0/18	0/18	4/18	3/18
**at age of 29 wks**	0/4	0/4	0/4	0/4
**at age of 35 wks**	0/7	0/7	2/7	1/7
**at age of 47 wks**	0/7	0/7	2/7	2/7
**Type 4; ascites (total)**	0/18	0/18	2/18	1/18
**at age of 29 wks**	0/4	0/4	0/4	0/4
**at age of 35 wks**	0/7	0/7	1/7	0/7
**at age of 47 wks**	0/7	0/7	1/7	1/7
**Type 5; infarction damage (total)**	2/18	2/18	5/18	4/18
**at age of 29 wks**	0/4	0/4	0/4	0/4
**at age of 35 wks**	1/7	1/7	2/7	2/7
**at age of 47 wks**	1/7	1/7	3/7	2/7

1. For hens died of sudden death, n=19, 12, 78, 57 in R, R+25-OH-D3, Ad, Ad+25-OH-D3 group, respectively.

2. For each group n=4, 7, 7 hens were sampled at age 29, 35 and 47 wks for tissue collection, total n=18 for each group.

3. Results of combinations of various cardiac pathological morphologies were shown in Table S2.

R; restriction, Ad; ad libitum, 25-OH-D3; 25-hydroxycholecalciferol.

Table S2. Effect of dietary supplementation of 25-OH-D3 on the combination of cardiac pathological morphologies of broiler breeder hens provided with restricted or ad libitum feed intake

Hens died by sudden[1, 3] (hearts of total dead hens)	Restriction	Restriction +25-OH-D3	Ad libitum	Ad libitum +25-OH-D3
**Type 1+3**	3/19	1/12	13/78	7/57
**Type 1+4**	1/19	0/12	4/78	2/57
**Type 1+5**	4/19	3/12	11/78	8/57
**Type 1+6**	1/19	0/12	2/78	0/57
**Type 2+3**	1/19	1/12	10/78	7/57
**Type 2+4**	1/19	1/12	3/78	2/57
**Type 2+5**	0/19	0/12	2/78	1/57
**Type 1+3+4**	0/19	0/12	2/78	1/57
**Type 1+3+5**	1/19	0/12	6/78	3/57
**Type 2+3+4**	1/19	1/12	3/78	2/57
**Hens necropsied for sampling[2, 3] (hearts of total sampling hens)**	**Restriction**	**Restriction +25-OH-D3**	**Ad libitum**	**Ad libitum +25-OH-D3**
**Type 1+3**	0/18	0/18	2/18	1/18
**Type 1+4**	0/18	0/18	1/18	1/18
**Type 1+5**	2/18	2/18	4/18	4/18
**Type 2+3**	0/18	0/18	2/18	1/18
**Type 2+4**	0/18	0/18	2/18	2/18
**Type 1+3+5**	0/18	0/18	1/18	1/18

1. For hens died by sudden death, n=19, 12, 78, 57 in R, R+25-OH-D3, Ad, Ad+25-OH-D3 group, respectively.

2. For each group n=4, 7, 7 hens were sampled at age 29, 35 and 47 wks for tissue collection, total n=18 for each group.

3. Type 1; concentric hypertrophy, Type 2; dilation, Type 3; pericardial effusion, Type 4; ascites, Type 5; infarction damage, Type 6; atrium rupture trauma.

R; restriction, Ad; ad libitum, 25-OH-D3; 25-hydroxycholecalciferol.

Table S3. Effect of dietary supplementation of 25-OH-D3 on the incidence of arrhythmic ECG patterns of broiler breeder hens provided with restricted or ad libitum feed intake.

	Restriction		Restriction +25-OH-D3		Ad libitum		Ad libitum +25-OH-D3	
**(hearts of sampling hens)**	**35 wk**	**47 wk**	**35 wk**	**47 wk**	**35 wk**	**47 wk**	**35 wk**	**47 wk**
**Normal**	5 / 7	6 / 7	5 / 7	5 / 7	2 / 7	3 / 7	3 / 7	3 / 7
**Type A**	0 / 7	0 / 7	0 / 7	0 / 7	0 / 7	0 / 7	0 / 7	1 / 7
**Type B**	0 / 7	1 / 7	0 / 7	0 / 7	0 / 7	1 / 7	1 / 7	1 / 7
**Type C**	0 / 7	0 / 7	1 / 7	0 / 7	0 / 7	0 / 7	0 / 7	0 / 7
**Type D**	0 / 7	0 / 7	0 / 7	0 / 7	1 / 7	1/ 7	1 / 7	1 / 7
**Type E**	2 / 7	0 / 7	0 / 7	1 / 7	0 / 7	0 / 7	0 / 7	0 / 7
**Type F**	0 / 7	0 / 7	1 / 7	1 / 7	1/ 7	1 / 7	1 / 7	0 / 7
**Type G**	0 / 7	0 / 7	0 / 7	0 / 7	2 / 7	0 / 7	1 / 7	1/ 7
**Type H**	0 / 7	0 / 7	0 / 7	0 / 7	1 / 7	1 / 7	0 / 7	0 / 7

Two days before sampling at 29, 35 and 47 wks for tissue collection, the selected hens (n=4, 7, and 7 hens for each group, respectively) were used for ECG measurement. No arrhythmic ECG patterns were observed at age of 29 wks.

Normal and specific arrhythmic ECG patterns (Type A to H) were shown in Figure S1

25-OH-D3; 25-hydroxycholecalciferol, ECG; electrocardiography.

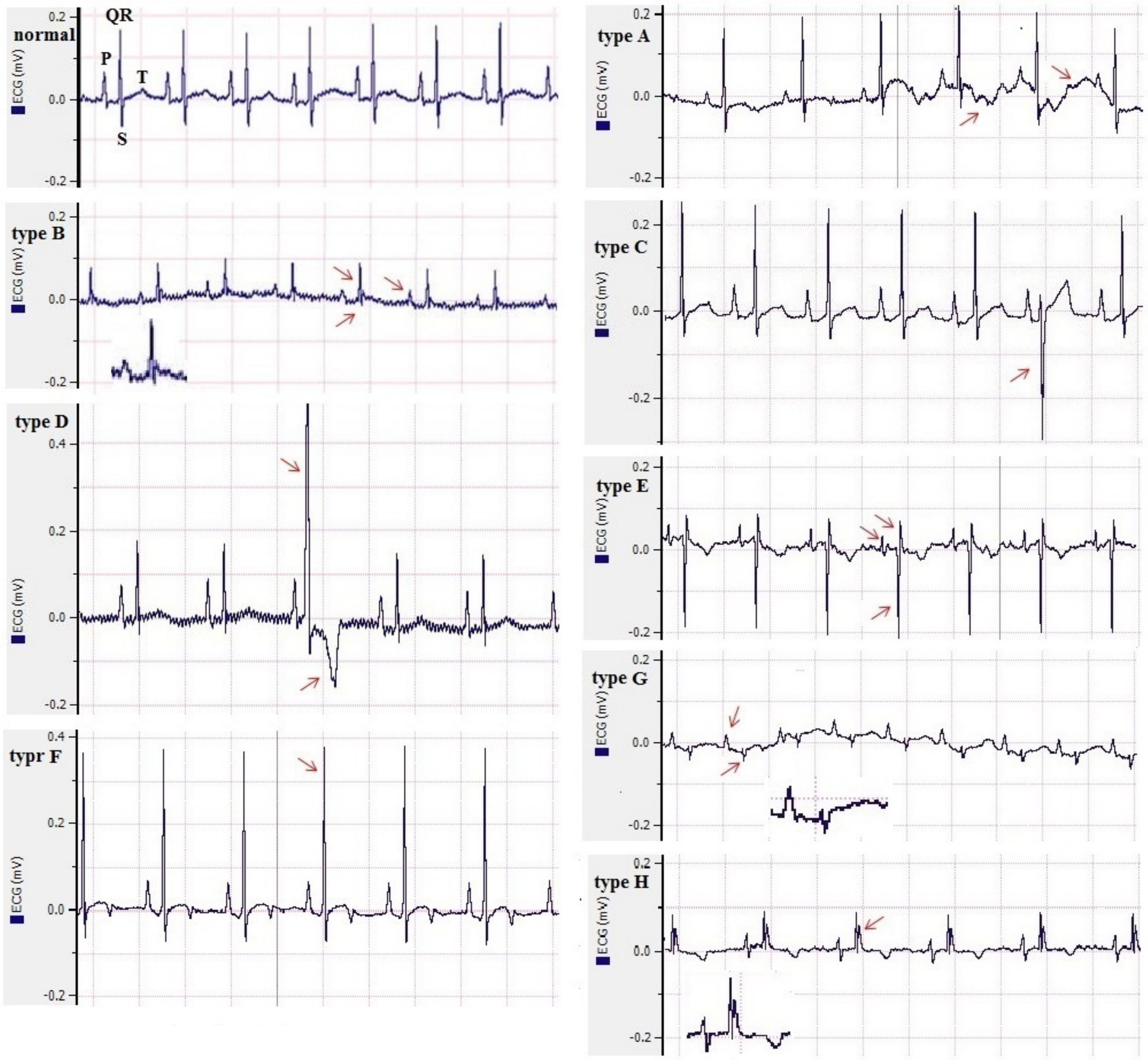

Figure S1. Effect of dietary supplementation of 25-OH-D3 on ECG pattern of broiler breeder hens provided with restricted or ad libitum feed intake.

Two days before sampling at 29, 35 and 47 wks for tissue collection, selected hens (n=4, 7, and 7 hens, respectively, for each group) were used for ECG measurement. Panel A-H; arrhythmic ECG patterns, arrows indicate irregularities. Panel A; irregular T wave, Panel B; a blunted P, QR, and T wave with the absence of S wave, Panel C; an irregular deep hyperpolarization of S wave, Panel D; an irregular extreme overshot of P wave following a deep hyperpolarization of T wave, Panel E; a suppressed amplitude of P and R wave with a known over-hyperpolarized Q wave, Panel F; an extreme overshoot of P wave, Panel G; an extremely blunted PQRST wave, Panel H; an Osborne wave after QR wave.

25-OH-D3; 25-hydroxycholecalciferol, ECG; electrocardiography.
